# Storage Stability of Texture and Sensory Properties of Yogurt with the Addition of Polymerized Whey Proteins

**DOI:** 10.3390/foods8110548

**Published:** 2019-11-04

**Authors:** Paulina Bierzuńska, Dorota Cais-Sokolińska, Asli Yiğit

**Affiliations:** 1 Department of Dairy Products Quality, Faculty of Food Science and Nutrition, Poznań University of Life Sciences, ul. Wojska Polskiego 31, 60-624 Poznań, Poland; cais@up.poznan.pl; 2 Department of Nutrition and Dietetics, Institute of Health Sciences, Marmara University, Başıbüyük Sağlık Yerleşkesi 9/3, 34854 Istanbul, Turkey; asliyigit8@hotmail.com

**Keywords:** yogurt, polymerized whey protein, texture, syneresis, sensory properties

## Abstract

Herein, we examined the possibility of producing probiotic yogurt with the addition of polymerized whey protein (PWP). It was determined that the yogurt was stable in terms of syneresis, texture, and sensory features. No spontaneous whey syneresis (SWS) was found in PWP yogurt during 21 days of refrigerated storage at 3 ± 0.5 °C. PWP yogurt had a 5.3% higher water retention capacity (WHC) than yogurt with whey protein concentrate (WPC). Compared with yogurt with unpolymerized protein, PWP yogurt had a higher absolute cohesiveness and viscosity index. The addition of whey protein concentrates to native and polymerized form resulted in longer maintenance of the original yogurt coherence than the control yogurt during storage. PWP yogurt had the same color saturation as the control yogurt. The polymerization of whey proteins resulted in a vanilla pudding aftertaste in yogurt and increased butter flavor 2.5-fold.

## 1. Introduction

Nowadays, consumers of dairy products, especially fermented milk, are increasingly demanding quality products, with pro-health and sensory features. Desirable sensory attributes include features such as appearance, texture, color, taste, and smell as well as whey flow. Moreover, these properties must maintain stability for the duration of the product shelf life. However, syneresis is considered the main disadvantage regarding the sensory attractiveness of yogurts. Syneresis is a consequence of shrinking milk protein gel, which decreases the size of casein aggregates promoting the separation of whey [1]. This often occurs during refrigerated storage of yogurt and is considered a technological defect. 

An effective solution is the use of polysaccharides, polyphenols, or whey protein concentrates (WPC) that bind water [2]. Polyphenols have the ability to interact with casein micelles generating a protein–polyphenol complex and thereby stabilizing the structure of yogurt. Dönmez et al. [2] examined the addition of green tea powder and green coffee powder as a source of polyphenols. The addition of 0.02% green tea powder and 2% green coffee powder significantly reduces yogurt syneresis during refrigerated storage. In order to improve the quality of yogurt, the water retention capacity must be enhanced, the production of a homogeneous structure, and high physical stability of the yogurt during storage must be achieved without adversely affecting the sensory characteristics. This can be accomplished using microbial transglutaminase. The reduction of syneresis is influenced by cross-linking microbial transglutaminase with milk proteins, thus stabilizing the three-dimensional structure of yogurt [3]. Vital et al. [1] proved the positive effect of inclusion in processing milk *Pleurotus ostreatus* aqueous extract to produce yogurt with attractive rheological, structural, and health-promoting properties. They showed that yogurt containing 1% *Pleurotus ostreatus* aqueous extract has the lowest syneresis, lower firmness but higher cohesiveness, adhesive, and springiness, and also increased antioxidant activity. Furthermore, it is important that the product not only provides health-promoting features but also attractive sensory qualities, with good consistency but above all lacks syneresis. According to Mahomud et al. [4], one of the methods to prevent whey separation in yogurt is the addition of whey protein concentrates. Generating soluble protein complexes improved physical, rheological, and microstructural properties.

Inspired by the above research, we examined the introduction of whey proteins in polymerized form in processing milk during the production of probiotic yogurt. Each heat treatment of proteins changes their structure, properties, and thus the ability to bind water. Our previous research has shown that yogurt with the addition of polymerized whey proteins (PWP) has up to several percent more antioxidant activity than yogurt with nonpolymerized whey proteins. In addition, the addition of PWP has a significant impact on the maintenance of the initial number of *Lactobacillus* bacteria during refrigerated storage [5]. Therefore, we explored how the PWP supplement shapes syneresis, appearance, texture, and sensory characteristics of yogurt. These features determine the acceptability of the product by the consumer.

## 2. Materials and Methods

### 2.1. Yogurt Preparation

The raw material used was commercial pasteurized cow’s milk (OSM, Głubczyce, Poland) with a solid non-fat (SNF) content of 9.07% and 1.50% fat. In the experiment, samples of yogurt were prepared from this milk: (1) Milk without additives; (2) The milk enriched with WPC80 (5.62% *w*/*v*), thus showing an increased dry matter (10.57%–16%); (3) The milk enriched with 28% PWP solution (*w*/*v*), thus showing an increased dry matter (10.57%–16%) [5].

The WPC80 (SM Spomlek, Radzyń Podlaski, Poland) contained 96.56% dry matter, including 79.43% proteins.

Preparation of the polymerized whey protein (28%, *w*/*v*) is as follows: WPC80 whey protein concentrate powder was dissolved in cold purified water and allowed to stand at 4 °C for 12 h. WPC dispersion was adjusted to pH 7.0 using 0.1 M sodium hydroxide at 21 °C. It was heated at 85 °C for 30 min and then rapidly cooled to room temperature in ice-water with agitation.

In the production of the yogurts, the starter culture used was a mixture of thermophilic bacteria, *Streptococcus thermophilus*, *Lactobacillus acidophilus*, and *Bifidobacterium animalis* subsp. *lactis*, with commercially available Lyofast SAB 440B from Sacco (Cadorago, Italy) being added at 10 units/25 L processed milk. Fermentation ran at 37 °C until pH 4.45 was obtained. A two-step cooling to 15 °C for a maximum of 15 min was applied, then the product was poured into unit containers of v = 150 mL and further cooled to 6 °C. Samples were produced on a pilot plant scale using factory-scale equipment (*n* = 24). They were tested 24 h after the completion of fermentation (day 0) and at 10 and 21 days of cold storage, i.e., at 3 ± 0.5 °C.

Compositional analysis and physicochemical properties of yogurt samples were determined as described by Bierzuńska and Cais-Sokolińska [5].

### 2.2. Determination of Water Holding Capacity

The water holding capacity (WHC) of yogurt is defined as its ability to hold all or part of its own water. WHC of the samples was determined using a slightly modified centrifugation method [6]. Yogurt (30 g) was centrifuged (model 260; MPW MED Instruments, Warsaw, Poland) under relative centrifugal force (RCF) = 10 732 g, rotor angle 30° (RPM 10 062 g) at 4 °C for 15 min. The supernatant was collected and weighed, and WHC was calculated according to the following equation:

WHC (%) = (1 − W_1_/W_2_)·100 (1)

where W_1_ is the weight in grams of the supernatant after centrifugation and W_2_ is the weight of the yogurt in grams.

### 2.3. Spontaneous Whey Syneresis

The siphon method described by Amatayakul et al. [7] was used with slight modifications to determine the extent of spontaneous whey syneresis (SWS) by Narayana and Gupta [8]. A 100 mL cup of yogurt was tilted 45° immediately after being removed from the refrigerator to collect the surface whey; this was siphoned off using a graduated syringe with a needle attached. The siphoning was performed within 10 s to avoid the forced leakage of whey from the curd.

### 2.4. Texture Measurement

The firmness, consistency, cohesiveness, and viscosity index of the fermented samples were determined using reverse extrusion in a TA-XTplus texture meter from Stable Micro Systems (Surrey, UK). The A/BE attachment with compression disc (Ø = 35 mm) was used. A sample was placed inside a cylinder with an internal diameter of Ø = 50 mm (75% filling). The measurement conditions were at distance 30 mm, pretest 1.0 mm/s and post-test 10.0 mm/s. Samples for analysis were prepared according to Cais-Sokolińska et al. [9]. Results were recorded in Texture Exponent E32 version 4.0.9.0 software (Godalming, Surrey, UK).

### 2.5. Color Measurement

The instrumental color measurement was based on the CIELab system described by Cais-Sokolińska et al. [10] A yogurt sample was placed in an OG optical glass cuvette 2/96G/10 (Starna Scientific Company Ltd, Ilford, UK). The measurement was performed with a D65 light source, and a 10° observation angle, with geometry SPIN using an SP-60 camera (X-Rite, Grandville, MI, USA) equipped with spherical geometry (diffusive), and the measurement chamber with a DRS-811 ceramic insert. The camera was calibrated based on the white and black reference standards SP-62-162 (X-Rite, Grandville, MI, USA). The chrome (C*), white index (WI), and yellowing index (YI) were calculated using equation:

C* = [(Δa*)^2^ + (Δb*)^2^]^0.5^ (2)

WI = [(ΔL)^2^ + (Δa*)^2^ + (Δb*)^2^]^0.5^ (3)

YI = 142.86b*·L^−1^ (4)

The calculations assumed: L = 100, a* = 0, and b* = 0.

### 2.6. Sensory Analysis

Sensors analysis was conducted via the profiling method [11,12]. Panel members (*n* = 14; 6 female, 8 male; aged between 21 and 52; Mage = 38.85, SD = 9.79; race White/Caucasian) were adequately trained individuals, prepared to perform sensors examinations [13,14]. Samples were evaluated using 8-cm unstructured line scales anchored with the terms low (denotes an undetectable points parameters) at the left and high (very intense) at the right. Sample temperature was 10–12 °C. The descriptors are listed in Table 1.

### 2.7. Statistical Evaluation

Verification of statistical hypotheses was accomplished by adopting an α = 0.05 level of significance. An ANOVA test was carried out, while for multiple post hoc comparisons, a Tukey HSD (Honestly Significant Difference) test was used. Dependent variables were the value of the parameters being studied, and independent variables were the type and time of the sample. The determination coefficient (R^2^) significance tests were based on the assumption of normal distribution of the residual value of the y variable and an equal residual value variation for all values of the x variable. The position of tested samples in the perception of the space results was evaluated using the Principal Component Analysis (PCA) based on the NIPALS algorithm. The statistical calculations were carried out using Statistical data analysis software, version 10 (StatSoft, Tulsa, OK, USA).

## 3. Results and Discussion

The total protein content increased 2.4-fold (*p* < 0.05) with the addition of whey protein, whereas in the case of casein maintained the same level (Table 2). Yogurt with WPC80 and PWP had less titratable acidity than the sample control (*p* < 0.05).

No spontaneous whey syneresis (SWS) was observed during refrigerated storage in PWP yogurt compared to the control sample and yogurt with WPC80 (Table 3). PWP yogurt displayed the highest WHC, which was 5.3% higher than WPC80 yogurt (*p* < 0.05; *p* = 0.0123; R^2^ = 0.616; WHC_PWP_ = 84.1243 + 0.1495·WHC_WPC80_). The mean WHC throughout the storage period of PWP yogurt was 96.69 ± 1.72 (mean ± SD; CV = 1.78; 5th to 95th percentile: 95.37 to 98.02). The ability of WPC80 yogurt to retain water during refrigerated storage decreased by about 20%, while PWP yogurt showed little difference.

Syneresis is an important index when evaluating yogurt quality. Fang and Guo [15] showed that the syneresis of the samples with PWP was lower compared to yogurt possessing unheated whey protein and significantly different toward the control yogurt. The obtained results showed that PWP yogurt had reduced syneresis compared to control yogurt. Wang et al. [16] showed that the addition of 0.4% PWP (*w*/*w*) and 0.3% pectin (*w*/*w*) to yogurt provides desirable consistency of yogurt and limits its syneresis. A similar result was reported by Li and Guo [17], where the incorporation of PWP increased viscosity by 80% and reduced the syneresis by 25%, and that PWP could act as a thickening agent that improves the rheological properties of yogurt. Mahomud et al. [4] determined that the addition of whey protein concentrates may prevent syneresis. They also proved that yogurt with the addition of heated skim milk and 2% WPC (*w*/*v*) had significantly higher storage modulus, water holding capacity, and firmness values, as well as a denser microstructure than those prepared only from skim milk.

The WPC80 add-on increased the firmness parameter value at each stage of storage (*p* < 0.05), but incorporation in polymerized form showed no difference compared to control yogurt (*p* > 0.05) (Table 4). Regardless of whether WPC80 was introduced in polymerized or nonpolymerized form, their consistency was significantly greater than that of control yogurt (*p* < 0.05). The absolute value of the cohesiveness parameter was the highest for PWP yogurt and lowest for WPC80 yogurt, regardless of storage time. The absolute value of the viscosity index was the highest for PWP yogurt. These values were significantly higher than the control yogurt, and even more so with WPC80 yogurt, which was 3-fold lower (*p* < 0.05). The addition of WPC80 and PWP caused longer maintenance of the original cohesiveness of yogurt than the control yogurt during storage. Storing yogurt with PWP and WPC80 showed a significant increase in consistency after 10 days of storage (*p* < 0.05), however, after 21 days no significant differences were observed compared to immediately after manufacturing (*p* > 0.05). This also applied to the viscosity index parameter. Firmness remained stable during storage of the tested yogurts (*p* > 0.05).

Denaturation of the globular whey proteins caused unfolding of their structure, which increased surface area. This exposed the buried peptides and amino acid side chains and increased the interaction with water and viscosity [18]. Fang and Guo [15] showed that the addition of PWP to yogurt resulted in higher viscosity than yogurt without PWP. The study conducted by Herrero and Requena [19] showed that supplementation of goat’s milk with WPC increased yogurt firmness and provided similar values to that of yogurt made form cow’s milk. They suggested that the increase in firmness of the yogurt with addition of WPC could be attributed by protein aggregates, formed by the interaction of casein micelles and denatured whey proteins via intermolecular disulfide bonds. Gustaw [20] examined the effect of whey protein addition on yogurt texture parameters, where whey protein aggregates generated by single heating had a more positive influence on the rheological properties of yogurt than those obtained by double heating. They also showed that yogurt hardness increased with longer whey protein aggregation times. Gustaw et al. [21] showed that yogurt produced with 2% double-heated whey protein isolate (WPI) had the highest apparent viscosity, which was 500 mPa∙s at a shear rate of 50/s, compared with yogurt possessing WPC and skimmed milk powder (118 mPa∙s and 200 mPa∙s, respectively).

WPC80 yogurt had the largest yellowness index (YI) (*p* < 0.05) (Table 5). The control yogurt and PWP yogurt had similar yellowness index (*p* > 0.05). PWP yogurt was furthest from the ideal white pattern compared to WPC80 yogurt (*p* < 0.05) only after manufacturing. The distance from the ideal white PWP yogurt pattern decreased with storage time (*p* < 0.05). During storage, PWP yogurt became more white. The opposite was true for WPC80 yogurt. However, regardless of storage time, PWP yogurt had the same saturated color as the control and 1.4-fold lower color saturation than nonpolymerized whey protein.

Based on the color measurements, it was possible to optimize and select the conditions of the technological process. A sensory evaluation of the brightness and color of dairy powders often did not reflect the differences found during instrumental analysis.

**Figure 1:**
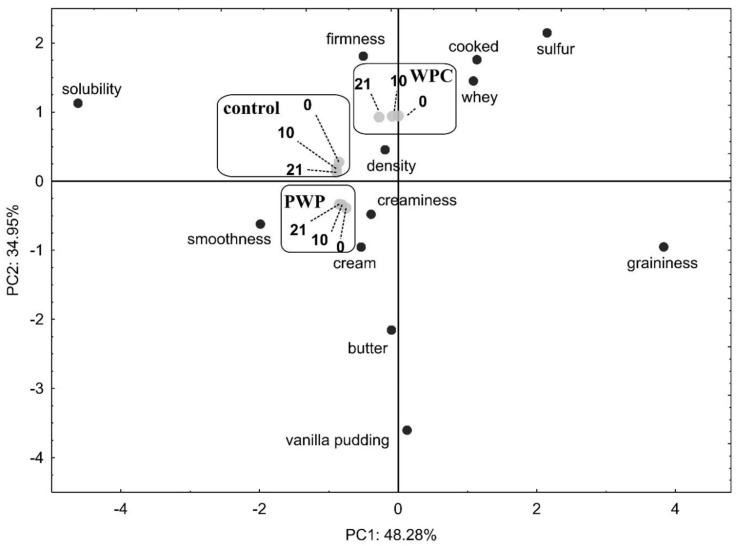
Principal component analysis biplot of sensory parameters used to differentiate yogurt with increased whey proteins content, including whey proteins after the polymerization process. 0, 10, 21, days of cold storage; WPC, yogurt with whey protein concentrate; PWP, yogurt with polymerized whey protein; PC, principal component.

PWP yogurt was characterized by more palpable cooked whey and sulfur than the yogurt control, but less than yogurt with WPC80 (Figure 1). Graininess did not matter in the characteristics of the samples. Vanilla pudding and butter, as well as smoothness, cream, and creaminess, were the most noticeable in PWP yogurt. It did not tend to change flavor, texture, or mouthfeel during storage. However, during storage of WPC80 yogurt, the perceptibility of whey and sulfur decreases while maintaining density. Polymerization caused lowering of cooked, whey, sulfur, intensification of cream, creaminess, smoothness, increasing butter, and vanilla pudding. Polymerization significantly influenced solubility, density, and firmness. Flavor vanilla pudding was not present in the control and WPC80 samples. In PWP yogurt, the mean flavor vanilla pudding throughout the storage period was 8.07 ± 0.16 (mean ± SD; CV = 1.97; 5th to 95th percentile: 8.02 to 8.12). After polymerization, the flavor of butter intensified 2.5-times.

There are few scientific reports that examine the effect of polymerization of whey protein concentrates on the sensory properties of yogurt. Fang and Guo [15] characterized the sensory characteristics of yogurt and proved that PWP yogurt had comparable sensory and textural characteristics to low-fat yogurt. In addition, they stated that PWP could be used as a fat replacement to develop low-fat yogurt with the desired properties.

## 4. Conclusions

The polymerization of whey proteins added to yogurt results in greater capacity for water retention. Thus, it significantly reduces the syneresis of yogurt during refrigerated storage. PWP yogurt compared to yogurt with native WPC had less firmness but higher absolute cohesiveness and viscosity index. The polymerization of whey proteins added to yogurt did not affect its instrumental measurement consistency. PWP yogurt had the same YI and C index as the control yogurt. The addition of unpolymerized whey proteins caused an increase in YI index, and C and WI compared to yogurt control. Polymerization may stop the process of yogurt color changes during storage. The polymerization process of whey proteins added to yogurt reduces the flavor of cooked, whey, sulfur-sensory yogurt. The addition of PWP to yogurt intensified palpability: cream, creaminess, smoothness, and butter growth. The aftertaste post-polymerization of the added yogurt proteins was flavor vanilla pudding.

## Figures and Tables

**Table 1:** Sensory attributes and description used to characterize the probiotic yogurt with polymerized whey protein (PWP).

Attribute Type and Attributes	Description
Flavor	
cooked	aromatics associated with cooked milk
whey	aromatics associated with cheddar cheese whey
sulfur	aromatics associated with sulfurous compounds
cream	intensity of raw cream aroma
butter	intensity of butter flavor
vanilla pudding	odor typical of vanilla pudding
Texture and mouthfeel	
solubility	property of a sample that quickly melts in the mouth
firmness	perceived firmness of the sample evaluated in the mouth
density	the thickness of the samples in the mouth after the panelists had taken a bite
creaminess	a velvety or soft feeling in the mouth (not fatty or oily)
smoothness	the extent to which the samples had an even consistency (absence of any granules)
graininess	perceived graininess of the sample evaluated in the mouth

**Table 2:** Composition and physicochemical characteristics of yogurt with polymerized whey protein.

Parameters	Control	With WPC80	With PWP
Solid non fat (g/kg)	91.2 ± 0.4 ^a^	159.1 ± 0.1 ^b^	161.0 ± 0.2 ^b^
TP (g/kg)	33.45 ± 0.02 ^a^	79.6 ± 0.01 ^b^	79.6 ± 0.03 ^b^
C (g/kg)	26.83 ± 0.01 ^a^	27.15 ± 0.05 ^a^	26.89 ± 0.01 ^a^
WP (g/kg)	5.62 ± 0.01 ^a^	8.54 ± 0.07 ^b^	8.51 ± 0.07 ^b^
Fat (g/kg)	15.1 ± 0.1 ^a^	15.0 ± 0.2 ^a^	15.0 ± 0.1 ^a^
Titratable acidity	0.875 ± 0.006 ^b^	0.853 ± 0.003 ^a^	0.850 ± 0.003 ^a^
pH	4.45 ± 0.02 ^a^	4.45 ± 0.01 ^a^	4.45 ± 0.01 ^a^

WPC80, yogurt with whey protein concentrate; PWP, yogurt with polymerized whey protein; TN, total nitrogen; NPN, non-protein nitrogen; TP, total protein (TN − NPN)·6.38; NCN, non-casein nitrogen; C, casein (TN − CN − NPN)·6.38; WP, whey protein (NCN − NPN)·6.38. Values represent mean ± standard deviation (*n* = 8). Titratable acidity is expressed as percentage of lactic acid. Different small letters in superscript in rows indicate statistically significant differences at the level α = 0.05.

**Table 3:** Syneresis of yogurt with increased whey proteins content, including whey proteins after the polymerization process.

Yogurt	Storage (d)	pH	SWS (%)	WHC (%)
control (*n* = 24)	0	4.45 ± 0.02 ^a^	0.1 ± 0.1 ^a^	95.23 ± 0.29 ^de^
	10	4.45 ± 0.01 ^a^	0.9 ± 0.6 ^b^	91.50 ± 1.01 ^be^
	21	4.42 ± 0.03 ^a^	1.5 ± 0.0 ^b^	87.54 ± 0.74 ^bc^
with WPC80 (*n* = 24)	0	4.45 ± 0.02 ^a^	0.1 ± 0.1 ^a^	92.41 ± 0.51 ^e^
	10	4.44 ± 0.01 ^a^	1.5 ± 0.4 ^b^	87.10 ± 3.25 ^c^
	21	4.42 ± 0.03 ^a^	3.9 ± 0.3 ^c^	72.63 ± 1.50 ^a^
with PWP (*n* = 24)	0	4.44 ± 0.02 ^a^	0.0 ± 0.0 ^a^	97.70 ± 0.82 ^d^
	10	4.44 ± 0.04 ^a^	0.0 ± 0.0 ^a^	97.57 ± 0.72 ^d^
	21	4.42 ± 0.03 ^a^	0.0 ± 0.0 ^a^	94.81 ± 1.65 ^de^

d, day; SWS, spontaneous whey syneresis; WHC, water holding capacity. Values represent mean ± standard deviation (*n* = 8). Different small letters in superscript in columns indicate statistically significant differences at the level α = 0.05.

**Table 4:** Texture parameters of yogurt with increased whey proteins content, including whey proteins after the polymerization process.

Yogurt	Storage (d)	Firmness (g)	Consistency (g∙s)	Cohesiveness |(g)|	Viscosity Index |(g∙s)|
control (*n* = 24)	0	34.21 ± 0.02 ^a^	961.37 ± 0.06 ^b^	43.50 ± 0.03 ^d^	81.23 ± 0.04 ^c^
	10	40.29 ± 0.01 ^a^	804.40 ± 0.01 ^a^	34.36 ± 0.02 ^c^	108.22 ± 0.02 ^d^
	21	36.64 ± 0.04 ^a^	805.11 ± 0.02 ^a^	34.00 ± 0.05 ^c^	79.65 ± 0.05 ^c^
with WPC80 (*n* = 24)	0	44.93 ± 0.04 ^b^	1003.27 ± 0.03 ^c^	24.43 ± 0.03 ^b^	40.10 ± 0.06 ^a^
	10	46.21 ± 0.06 ^b^	1089.05 ± 0.01 ^d^	24.57 ± 0.04 ^b^	45.93 ± 0.04 ^b^
	21	44.57 ± 0.01 ^b^	1029.01 ± 0.05 ^c^	19.07 ± 0.01 ^a^	41.99 ± 0.05 ^a^
with PWP (*n* = 24)	0	36.50 ± 0.03 ^a^	1067.02 ± 0.04 ^c^	56.71 ± 0.05 ^f^	139.99 ± 0.06 ^e^
	10	39.36 ± 0.05 ^a^	1161.20 ± 0.06 ^d^	54.71 ± 0.07 ^f^	147.72 ± 0.03 ^f^
	21	35.29 ± 0.01 ^a^	1057.28 ± 0.07 ^c^	45.36 ± 0.01 ^e^	125.83 ± 0.02 ^e^

Values represent mean ± standard deviation (*n* = 8). Different small letters in superscript in columns indicate statistically significant differences at the level α = 0.05.

**Table 5:** Assessment of the color of yogurt with increased whey proteins content, including whey proteins after the polymerization process.

Yogurt	Storage (d)	WI	YI	C*
control (*n* = 24)	0	10.38 ± 0.41 ^b^	12.18 ± 0.21 ^a^	8.17 ± 0.18 ^b^
	10	8.76 ± 0.12 ^a^	11.82 ± 0.21 ^a^	8.17 ± 0.18 ^b^
	21	8.28 ± 0.40 ^a^	11.42 ± 0.64 ^a^	7.96 ± 0.44 ^b^
with WPC80 (*n* = 24)	0	15.44 ± 1.76 ^d^	18.18 ± 0.63 ^b^	11.41 ± 0.47 ^c^
	10	17.11 ± 0.73 ^e^	18.65 ± 0.18 ^b^	11.39 ± 0.12 ^c^
	21	17.10 ± 1.64 ^e^	18.86 ± 0.15 ^b^	11.54 ± 0.30 ^c^
with PWP (*n* = 24)	0	19.92 ± 2.53 ^e^	12.54 ± 0.24 ^a^	7.27 ± 0.11 ^a^
	10	18.71 ± 0.98 ^d^	13.68 ± 0.22 ^a^	8.08 ± 0.05 ^b^
	21	12.75 ± 0.53 ^c^	13.21 ± 0.22 ^a^	8.50 ± 0.15 ^b^

WI, white index; YI, yellowing index; C*, chrome. Values represent mean ± standard deviation (*n* = 8). Different small letters in superscript in columns indicate statistically significant differences at the level α = 0.05.
